# Enterovirus D68 infection among hospitalized children with severe acute respiratory illness in El Salvador and Panama, 2012‐2013

**DOI:** 10.1111/irv.12815

**Published:** 2020-12-05

**Authors:** Holly M. Biggs, W. Allan Nix, Jing Zhang, Shannon Rogers, Wilfrido Clara, Jorge H. Jara, Rosalba Gonzalez, Kathia Luciani, Yarisa Sujey Brizuela, Dora Estripeaut, Juan Miguel Castillo, Tirza De Leon, Mary Corro, Ofelina Vergara, Rafael Rauda, Evens G. Chong, John T. Watson, Eduardo Azziz‐Baumgartner, Susan I. Gerber, Suxiang Tong, Fatimah S. Dawood

**Affiliations:** ^1^ National Center for Immunization and Respiratory Diseases Centers for Disease Control and Prevention Atlanta GA USA; ^2^ Centro de Estudios en Salud Universidad del Valle de Guatemala Guatemala City Guatemala; ^3^ Gorgas Memorial Institute for Health Studies Panama City Panama; ^4^ Hospital De Especialidades Pediátricas Omar Torrijos Panama City Panama; ^5^ Hospital San Juan De Dios San Miguel El Salvador; ^6^ Hospital Del Niño Panama City Panama; ^7^ Hospital San Juan De Dios Santa Ana El Salvador; ^8^ Hospital Materno Infantil José Domingo De Obaldía David Panama

**Keywords:** acute respiratory illness, Central America, El Salvador, Enterovirus D68, Panama, respiratory virus

## Abstract

We assessed EV‐D68 epidemiology and phylogenetics among children aged ≤9 years hospitalized with severe acute respiratory illnesses at five sites in Panama and El Salvador during 2012‐2013. Respiratory specimens positive for enterovirus or rhinovirus were tested by real‐time RT‐PCR for EV‐D68, and partial VP1 gene sequences were determined. Of 715 enrolled children, 17 from sites in both countries were EV‐D68‐positive and commonly had a history of asthma or wheezing. Phylogenetically, 15 of 16 sequences fell into Clade B1, and one into Clade A2. The Central American EV‐D68s were closely related genetically to contemporaneous strains from North America, South America, and the Caribbean.

## 1 INTRODUCTION

From the first description of enterovirus D68 (EV‐D68) in 1962 until 2014, sporadic cases and small clusters of EV‐D68 respiratory disease were reported from around the globe. During 2014, a large outbreak of EV‐D68 occurred in the United States and Canada, with smaller numbers of cases reported in subsequent years.^1^, ^2^, ^3^ Few cases of EV‐D68 are reported in the literature from Latin America before 2014.^4^, ^5^ During 2014, an outbreak of EV‐D68 was reported from Mexico City; however, a widespread epidemic, such as occurred in the United States, was not documented.^6^ Additionally, during 2014, two EV‐D68 cases were reported from Chile and four from two Caribbean islands.^7^, ^8^

Since 2014, known circulating EV‐D68 strains fall into two clades: A and B, with Clade A divided into subclades 1 and 2 and Clade B divided into subclades 1‐3. During 2014, Clade B1 was the predominant lineage detected from the United States and Canada, as well as from a limited number of viruses characterized from Mexico, Chile, and the Caribbean.^1^, ^3^, ^6^, ^7^, ^8^ Clade B1 had also been documented in the United States before the 2014 outbreak, during 2011‐2013.^1^ In years subsequent to 2014, Clade B1 has been less commonly detected in the United States, with Clade B3 predominating most recently^9^, ^10^ and also detected from Argentina during 2016.^11^

Overall, little is known about EV‐D68 circulation, epidemiology, and phylogenetics in Central America. We evaluated EV‐D68 infection among children hospitalized with severe acute respiratory illness in Panama and El Salvador during 2012‐2013.

## 2 METHODS

During September‐October 2012 and April‐October 2013, a multi‐site clinical trial evaluating empiric oseltamivir treatment of hospitalized children was conducted at three hospitals in Panama (two in Panama City and one in David) and two hospitals in El Salvador (one in Santa Ana and one in San Miguel; with the San Miguel site enrolling during 2013 only).^12^ Children were eligible for enrollment if they were aged ≤9 years and hospitalized <7 days after symptom onset with symptoms meeting a modified version of the World Health Organization criteria for severe acute respiratory infection (cough or sore throat plus age‐specific tachypnea). Study hospitals were tertiary care facilities that provided specialized pediatric care. Enrollment periods were selected to coincide with periods of influenza virus circulation. At enrollment, study physicians conducted a baseline clinical assessment, collected mid‐turbinate nasal and throat swabs (or endotracheal aspirates for intubated children), and administered a questionnaire to the parent or guardian on participant demographics, medical history and history of illness.

Respiratory specimens were stored and tested at the Gorgas Memorial Institute for Health Studies by conventional singleplex real‐time RT‐PCR (rRT‐PCR) using CDC protocols for influenza A and B viruses, respiratory syncytial virus, parainfluenza viruses 1‐3, human metapneumovirus and rhinovirus. Specimens negative for all viruses by conventional singleplex rRT‐PCR were tested by a respiratory panel pan‐viral family/genus PCR (including pan enterovirus PCR) at CDC.^13^ Enterovirus or rhinovirus positive specimens, by either pan enterovirus PCR or rhinovirus rRT‐PCR, were tested for EV‐D68 using a CDC‐developed EV‐D68‐specific rRT‐PCR assay.^14^ For EV‐D68‐positive specimens, partial viral protein 1 (VP1) region sequences were determined.^15^ and analyzed with MEGA6.0,^16^ using the neighbor‐joining method to infer the evolutionary history.^17^

We describe frequencies of demographic and clinical characteristics of children with EV‐D68 infection and all enrolled children with respiratory specimens tested for enterovirus/rhinovirus. Selected characteristics of children with and without EV‐D68 infection were compared using Pearson's chi‐square, Fischer's exact test, or Wilcoxon rank‐sum test as appropriate. All p‐values were 2‐sided and evaluated for statistical significance at *P* < .05. Data were analyzed using STATA version 14 (STATACorp, College Station, TX).

The study protocol was approved by the national ethical review committees of El Salvador and Panama, upon which the CDC ethical review committee relied.

## 3 RESULTS

Seventeen EV‐D68 infections were identified among 715 children enrolled with respiratory specimens tested (Table 1). Sixteen of the 17 children with EV‐D68 infection were enrolled at two sites: San Miguel, El Salvador and David, Panama. Infections occurred predominantly during August‐October 2013, with one infection detected during June 2013. EV‐D68 was not detected among 63 children enrolled during September‐October 2012 (Figure S1).

**Table 1:** Demographics, Clinical Characteristics, and Outcomes of Enterovirus D68 (EV‐D68)‐Positive and All Enrolled Children, Panama and El Salvador, 2012‐2013, N = 715

	EV‐D68‐Positive N = 17 n (%)	All Enrolled N = 715 n (%)
Age — y		
Median	2.3^*^	1.0
Range	0.6‐9	0.02‐9.6
Age group		
0 to <6 mo	0	188 (26)
6 mo to <1 y	3 (18)	179 (25)
1 to <2 y	3 (18)	185 (25)
2 to <5 y	6 (35)	128 (18)
5 to <10 y	5 (29)^*^	35 (5)
Sex		
Male	8 (47)	424 (59)
Female	9 (53)	291 (41)
Country		
Panama	7 (41)	325 (45)
Panama City (Site 1)	0	159 (22)
Panama City (Site 2)	0	79 (11)
David	7 (41)	87 (12)
El Salvador	10 (59)	390 (55)
Santa Ana	1 (6)	169 (24)
San Miguel^†^	9 (53)	221 (31)
Medical History‡		
Any underlying condition	9 (53)^*^	161 (23)
Asthma	9 (53)^*^	131 (18)
Bronchopulmonary dysplasia	0	5 (1)
Chronic cardiovascular disease	0	12 (2)
Family history of asthma	7 (41)	278 (39)
Symptoms (parent report)		
Cough	17 (100)	709 (99)
Rhinorrhea	17 (100)	632 (88)
Difficulty breathing	16 (94)	627 (88)
Wheezing	15 (88)	461/698 (66)
History of wheezing with previous illness^§^	14/15 (93)^*^	255/459 (56)
Fever	12 (71)	557 (78)
Decreased appetite	6 (35)	307 (43)
Vomiting	5 (29)	158 (22)
Chest pain	3 (18)^*^	29/602 (5)
Diarrhea	3 (18)	74 (10)
Sore throat	3 (18)	45/617 (7)
Abdominal pain	2 (12)	23/695 (3)
Chills	1 (12)	56/711 (8)
Earache	0	8/633 (1)
Drowsiness	0	54 (8)
Difficult to wake	0	25 (4)
Illness duration before admission — days		
Median	2	3
Interquartile range	1‐3	2‐4
Fever ≥ 38.0 Celsius at admission	1 (6)	85 (12)
Hypoxia at admission^¶^	2 (12)	63 (9)
Oxygen requirement at admission	2 (12)^*^	267 (37)
Mechanical ventilation at admission	1 (6)	6 (1)
Increased work of breathing^e^	16 (94)	594 (83)
Retractions	16 (94)	588 (82)
Nasal flaring	3 (18)	119 (17)
Grunting	0	29 (4)
Lung exam by study physician		
Wheezing	15 (88)	517 (73)
Rhonchi	12 (71)	394 (56)
Rales	8 (47)	462 (65)
Decreased breath sounds	1 (6)	20 (3)
Normal	0	6 (<1)
Chest radiograph performed^f^	13 (76)	572 (80)
Radiographic pneumonia		
Yes	10/13 (77)	376/572 (66)
No	2/13 (15)	122/572 (21)
Not recorded	1/13 (8)	74/572 (13)
Died	0	1 (<1)

^e^ Presence of ≥1 of supraclavicular retractions, subcostal or intercostal retractions, nasal flaring, grunting, or need for noninvasive or invasive mechanical ventilation.

^f^ Chest radiograph performed in emergency department or during first day of admission. Radiographically confirmed pneumonia included chart documentation of a chest radiograph interpreted by either the treating physician or radiologist as showing pneumonia.

* Significant difference (*P* < .05) between children with and without EV‐D68‐infection (not assessed for enrollment site variable).

^†^ Enrolled during 2013 only.

^‡^ Other medical conditions (n < 5 for each) include “other chronic lung disease,” hemoglobinopathy, “other metabolic disorder,” developmental delay, seizure disorder, and neuromuscular disorder.

^§^ Asked only for patients with parent‐reported wheezing with current illness.

^¶^ Oxygen saturation < 92% measured by pulse oximetry while breathing room air, or need for non‐invasive or invasive mechanical ventilation.

Among the 17 EV‐D68‐positive children, the median age was 2.3 (range 0.6‐9) years and eight (47%) were male. Children with EV‐D68 infection were older than the overall study population for which the median age was 1.0 (range 0.02‐9.6) years (*P* < .001). A history of asthma in nine (53%) children was the only underlying condition reported among the 17 with EV‐D68 infection. Asthma was more common among children with EV‐D68 infection (53% vs 18%, *P* = .001).

All 17 children with EV‐D68 infection had a history of cough and rhinorrhea, 16 (94%) had difficulty breathing, 15 (88%) had wheezing, and 12 (71%) had a history of fever. Among the 15 children with reported wheezing, 14 (93%) had a history of asthma or a previous illness with wheezing. At enrollment, only one (6%) EV‐D68‐positive patient had a measured fever (temperature ≥ 38°C), although six (35%) had received an antipyretic within the past 24 hours. Two (12%) patients had hypoxia, including one with an oxygen saturation of 91% on room air and one requiring mechanical ventilation. Among 13 children with a chest radiograph performed, ten (77%) had radiograph‐confirmed pneumonia based on interpretation by the treating clinician or radiologist. One patient who required mechanical ventilation was admitted to the intensive care unit. No children with EV‐D68 infection died. The median number of days hospitalized was three (range 1‐14). Primary discharge diagnoses included pneumonia (n = 11, 65%), asthma exacerbation (n = 3, 18%), and bronchiolitis or bronchitis (n = 3, 18%).

**Figure 1:**
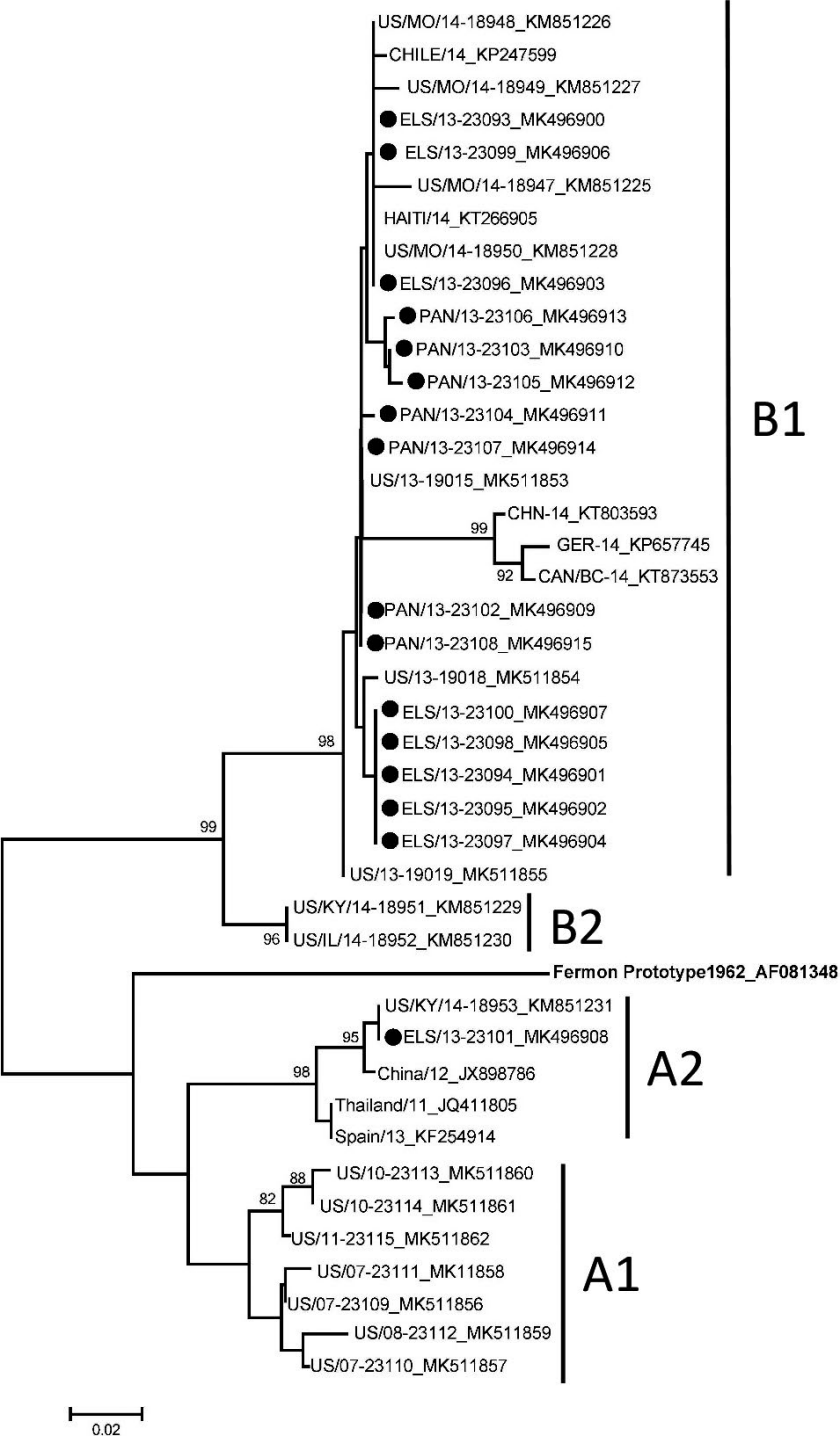
Phylogenetic tree including enterovirus D68 (EV‐D68) sequences from El Salvador and Panama. Using EV‐D68 partial viral protein 1 sequences, the evolutionary history was inferred using the neighbor‐joining method in MEGA 6.0^16^, ^17^; contemporaneous EV‐D68 reference strains from GenBank were included in the analyses. EV‐D68s on the tree are designated by country/state or province for some/year of detection‐strain name_GenBank accession number. Solid triangles represent sequences from El Salvador (n = 9), and solid circles represent sequences from Panama (n = 7). Bootstrap values >80% are shown. The scale bar represents genetic change in base substitutions per site

Sixteen of 17 EV‐D68‐positive samples were sequenced (GenBank accession numbers MK511853‐MK511862 and MK496900‐MK496915). Fifteen VP1 sequences fell into Clade B1 and one into Clade A2 (Figure 1). The single Clade A2 sequence was from San Miguel, El Salvador, and was detected during October 2013, during the same time that Clade B1 viruses were detected from the same site. The Central American EV‐D68 clade B1 strains share 98.2%‐100% nucleotide identity with each other, with US strains (2013 and 2014) and with 2014 strains from Haiti and Chile. The single clade A2 EV‐D68 from El Salvador shares 98.2%‐100% nucleotide identity with strains from the United States (2014), Asia (2011 and 2012), and Europe (2013).

## 4 DISCUSSION

We identified EV‐D68 infection in 17 children hospitalized with severe acute respiratory illness in Panama and El Salvador during 2013. The predominant clade, B1, was the same clade detected most commonly from the EV‐D68 outbreak in the United States and Canada, as well as from Mexico, Chile, and the Caribbean during 2014,^1^, ^3^, ^6^, ^7^, ^8^ and that also circulated in the United States during 2011‐2013.^1^ Clade A2, identified from one child in our study, was reported rarely in the United States during the 2014 outbreak and has continued to circulate with a small number of detections in the United States during 2017 and 2018 (CDC Picornavirus Laboratory).^1^, ^18^ Reports describing circulation of enteroviruses, and in particular EV‐D68, in Central America are limited.^4^ EV‐D68 likely circulates endemically around the globe, with occasional epidemic years with unknown triggers and some reports of a biennial pattern.^19^ No reports of EV‐D68 outbreaks from El Salvador or Panama emerged during the 2014 US outbreak or subsequently; however, lack of clinical laboratory testing availability may have limited detection.

Data on enterovirus seasonal trends in Central America are scarce,^4^ and it is unknown whether seasonal patterns of EV‐D68 circulation in Central America are similar to those observed in the United States; however, most EV‐D68 infections in this study occurred during August—October, consistent with the expected seasonality in North America. The significance of detection of EV‐D68 at three rural sites without detection at the two urban Panama City site is unknown but may indicate regional differences in EV‐D68 circulation.

Consistent with other descriptions, children with EV‐D68 infection in our study commonly had wheezing and a history of asthma or wheezing with previous illnesses.^1^, ^20^ Although increased work of breathing was common, need for mechanical ventilation was uncommon and occurred in only one child. Among reported symptoms, no common symptom distinguished EV‐D68 infection from other severe acute respiratory illness, highlighting the importance of diagnostic tests in understanding EV‐D68 epidemiology.

This study had several limitations. Enrollment was limited to a seven‐month period coinciding with the anticipated influenza season, which might not coincide with the peak circulation of EV‐D68. Specimens that tested negative on the initial rhinovirus rRT‐PCR assay and had another pathogen identified were not routinely tested using the EV‐D68 specific assay; it is possible that some EV‐D68 infections could have been missed among children with another viral detection. All enrolled children had severe respiratory illness with age‐specific tachypnea; therefore, children with EV‐D68 illness without tachypnea would not have been captured. Asthma and prior wheezing history was ascertained by parent or guardian interview only.

In conclusion, EV‐D68 was associated with severe acute respiratory illness among children in El Salvador and Panama, including a high prevalence of asthma or prior wheezing among affected children. The clade most commonly detected was the same that predominated globally during 2014 and was closely related to strains identified in the United States, Asia, and Europe during 2011‐2013.^1^ Additional respiratory virus surveillance, including testing for EV‐D68, would improve understanding of circulation patterns in Central America over time.

## CONFLICTS OF INTEREST

All authors declare no conflicts.

## DISCLOSURES

The findings and conclusions in this report are those of the authors and do not necessarily represent the official position of the Centers for Disease Control and Prevention.

## AUTHOR CONTRIBUTION

**Holly M Biggs:** Formal analysis (equal); Writing‐original draft (equal). **W. Allan Nix:** Formal analysis (equal); Investigation (equal); Visualization (equal); Writing‐review & editing (equal). **Jing Zhang:** Investigation (equal). **Shannon Rogers:** Investigation (equal). **Alexey Clara:** Investigation (equal). **Jorge Jara:** Conceptualization (equal); Investigation (equal); Methodology (equal); Project administration (equal); Supervision (equal). **Rosalba Gonzalez:** Methodology (equal); Project administration (equal). **Kathia Luciani:** Investigation (equal). **Yarisa Sujey Brizuela:** Investigation (equal). **Dora Estripeaut:** Investigation (equal). **Juan Miguel Castillo:** Investigation (equal). **Tirza De Leon:** Investigation (equal). **Mary Corro:** Investigation (equal). **Ofelina Vergara:** Investigation (equal). **Rafael Rauda:** Investigation (equal). **Evens G Chong:** Investigation (equal). **John T. Watson:** Writing‐review & editing (equal). **Eduardo Azziz‐Baumgartner:** Conceptualization (equal); Writing‐review & editing (equal). **Susan I. Gerber:** Conceptualization (equal); Writing‐review & editing (equal). **Suxiang Tong:** Conceptualization (equal); Investigation (equal); Methodology (equal); Writing‐review & editing (equal). **Fatimah S Dawood:** Conceptualization (equal); Methodology (equal); Supervision (equal); Writing‐review & editing (equal).

### Peer Review

The peer review history for this article is available at https://publons.com/publon/10.1111/irv.12815.

## Supporting information

Fig S1
