# Supplementary material for: Enterovirus D68 infection among hospitalized children with severe acute respiratory illness in El Salvador and Panama, 2012‐2013
Source: Influenza Other Respir Viruses. 2020 Dec 5;15(2):181–7. doi: 10.1111/irv.12815 (PMC7902261; doi:10.1111/irv.12815)
Supplement: Supplementary file 1 — Fig S1 [file IRV-15-181-s001.docx]

**Supplemental Figure**. Enterovirus D68 among children with severe acute respiratory illness, weekly number positive by illness onset date, El Salvador and Panama
